# Geospatial mapping of malaria risk in flood-prone zones of Sub-Saharan Africa

**DOI:** 10.1038/s41598-026-54811-7

**Published:** 2026-06-10

**Authors:** Jeremy Eudaric, Marleen C. de Ruiter, Nivedita Sairam, Andrés Camero, Kasra Rafiezadeh Shahi, Mark W. Smith, Xiao Xiang Zhu, Heidi Kreibich

**Affiliations:** 1 Chair of Data Science in Earth Observation, Technical University of Munich, Munich, Germany; 2 German Aerospace Center, (DLR), Wessling, Germany; 3 Institute for Environmental Studies, Vrije Universiteit Amsterdam, Amsterdam, 1081HV the Netherlands; 4 Helmholtz Center for Geosciences, (Section Hydrology), Potsdam, Germany; 5 Potsdam Institute for Climate Impact Research, Postdam, Germany; 6 School of Geography and Water@Leeds, University of Leeds, Leeds, LS2 9JT UK; 7 Munich Center for Machine Learning, Munich, Germany

**Keywords:** Malaria, Flooding, Geospatial, Sub-Saharan Africa, Diseases, Ecology, Ecology

## Abstract

The World Health Organisation (WHO) aims to eliminate malaria by 2030; yet, the disease remains endemic in Sub-Saharan Africa. Stagnant floodwaters provide ideal breeding grounds for mosquitoes. Previous estimates of potential malaria risk in flood zones have been limited due to insufficient large-scale geospatial data. Here, we integrate high-resolution flood maps (2000–2018) from the Global Flood Database, malaria incidence data from the Malaria Atlas Project, and geospatial population data across 492 flood-prone zones in 38 countries. We used a geospatial statistical models to assess malaria relative risk and drivers. We found that in East and West Africa, malaria relative risk is elevated in flood-prone regions compared to national baselines. We estimate that $\sim$12 million individuals diagnosed with *Plasmodium falciparum (Pf* )were exposed to flooding events, representing one-third of the population affected by floods. Our analyses find that flood exposure is one of the main drivers of the malaria burden in flood zones. These findings identify critical malaria hotspots and key drivers in flood-prone zones, and can help inform WHO’s malaria eradication strategies by guiding policymakers on the geographic distribution of vulnerable areas.

## Introduction

Malaria is the most prevalent infectious disease in Sub-Saharan Africa^1^. WHO analysis shows that the African region bears the most significant burden of malaria, accounting for 94% of global cases and 95% of deaths, which translates to approximately 233 million cases and 580,000 fatalities worldwide^2^. Transmitted by *Anopheles mosquitoes*, *Plasmodium falciparum* is the most deadly species of malaria-causing parasite^3^. Changes in malaria transmission are complex and influenced by factors beyond climate alone^4^. The transmission pattern of the vector-borne disease can be associated with many drivers: social, demographic, environmental, climate, hydrology and economic factors^2,5–8^.

Floods are projected to increase due to perturbations and amplifications of the water cycle^9,10^. With climate change projected to intensify flooding and malaria risk, understanding how flood exposure modifies malaria risk is crucial for global health planning and adaptation strategies^11,12^. Exposure is the situation of people and infrastructure located in hazard zones. The proportion of people globally exposed to floods will increase^13^. 170 million people are at high risk for floods and extreme poverty, 44% are in Sub-Saharan Africa^14^.

Assessing the impact of floods on malaria risk is crucial in a warming world. Flooding can influence malaria transmission through multiple ecological mechanisms affecting vector breeding, survival, and habitat formation. Stagnant floodwaters and, in particular, receding floodwaters can create new aquatic habitats such as pools, puddles, and disconnected surface water bodies that serve as breeding grounds for *Anopheles* mosquitoes^15^. These habitats often exhibit favourable conditions for larval development, including increased water persistence, temperature, and sunlight exposure. At the same time, the relationship is not uniformly positive. Intense rainfall and flooding events can also reduce vector populations by flushing out larvae and destabilising breeding sites^15^. Consequently, the link between meteorological events such as rainfall and malaria is well established but highly nonlinear^8^. Floods have been observed to increase local malaria risk^16^, with peaks in malaria cases often occurring several weeks after floodwaters recede, when stable breeding habitats are most abundant^15^. In the future, the changing climate and the increase of flood events will increase malaria cases in Africa^17^.

**Fig. 1:**
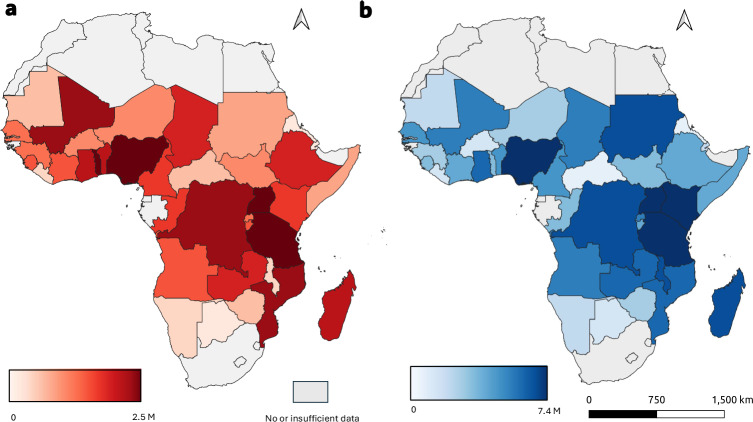
Malaria burden in the flood areas. **a, ** Estimation number of people diagnosed with *Pf*, exposed to flood events. **b,** Estimation of the number of people exposed to floods.

Despite evidence of local associations between floods and malaria outbreaks, a comprehensive, large-scale global assessment integrating high-resolution flood maps, population data, and malaria incidence is lacking^15,16^. Here, we use flood maps derived from the Global Flood Database (GFD)^13^ between 2000 and 2018, analysing 492 flood-prone zones, as shown in Fig. 1. These were combined with geospatial population data and diagnosed *Pf* cases from the Malaria Atlas Project^18^. Using geospatial analysis, we found that $\sim$ 12 million people exposed to flood events have been diagnosed with *Pf*. We first analyse the global mean relative malaria risk over the study period and investigate its temporal trends to quantify the relative malaria risk associated with each flood event. Second, we use a hierarchical Bayesianmodel to assess the key environmental and socioeconomic confounding factors that most strongly influence the *Pf* cases in the flood zones. This study provides a new roadmap for the WHO in its malaria eradication efforts by highlighting the influence of flood events on the potential malaria burden. The assessment offers a more straightforward overview of spatial hotspots and drivers necessary for public health fund deployment.

## Results

### Flood exposure and malaria burden

In sub-Saharan Africa, we observed that 12 million individuals diagnosed with *Pf* were exposed to flooding between 2000 and 2018 (Fig. 1 and Source Data Fig. 1). This count represents 33% of the total population exposed to floods. The countries with the highest numbers of new malaria cases in flood areas are Uganda (2.5 million), followed by Nigeria (1.8 million) and Mauritania (1.2 million) (Fig. 1, Source Data Fig. 1). The countries with the lowest numbers of cases exposed to floods are Djibouti, Botswana, and Eritrea. Within the African subregion, West and East Africa have the highest number of flood-exposed cases. In parallel, focusing on the number of people exposed to flood between 2000-2018, Kenya is the country with the highest.

### Statistical analyses

**Fig. 2:**
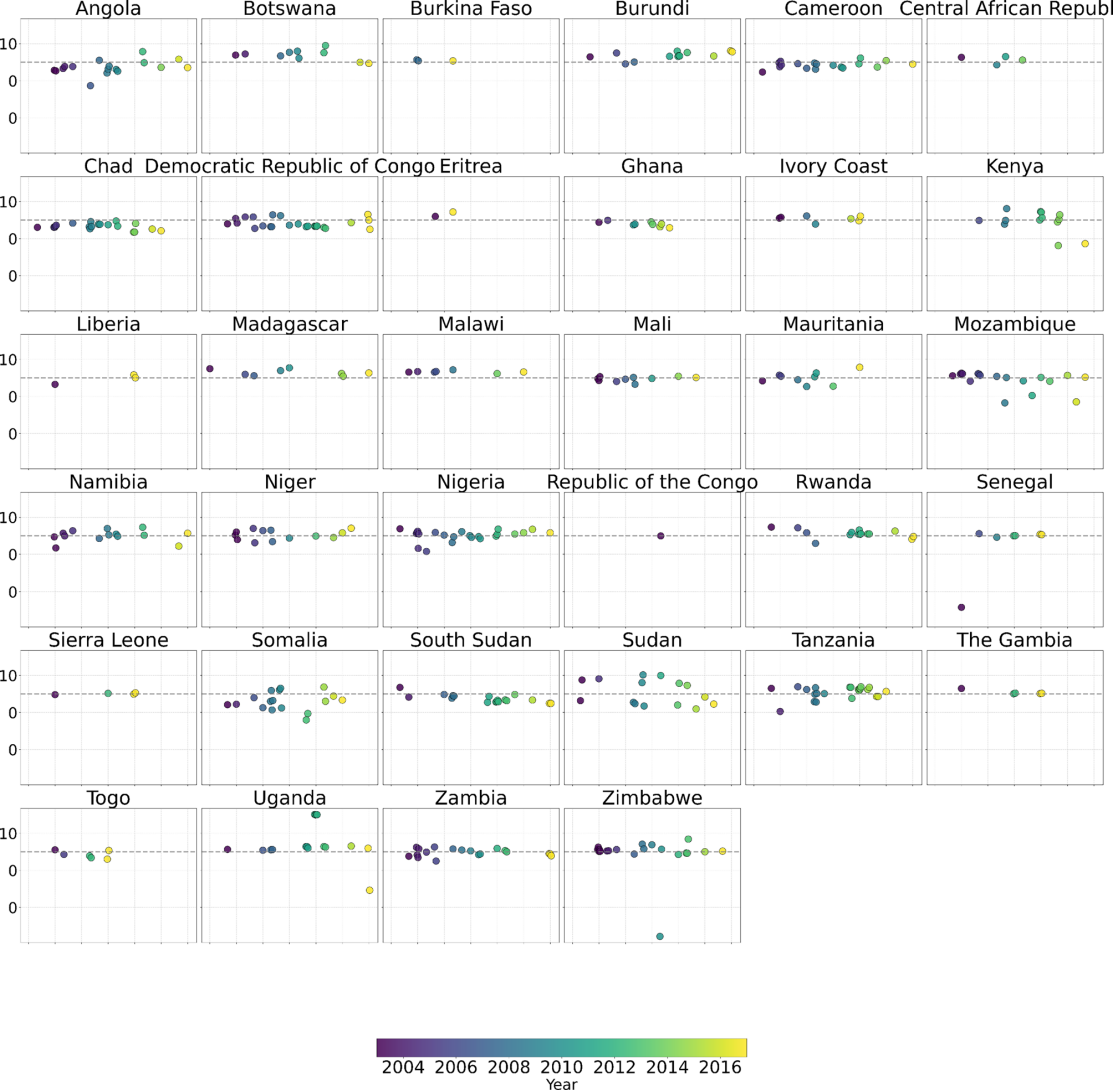
Change of the RR between 2000 and 2018. For each country and each flood event, we can observe the change in RR over time. The dots above the grey line represent an RR positif ($RR > 1$) and below an RR negatif ($RR < 1$). Nineteen countries hold all the dominant points above the reference line, with a positive RR, indicating a deviation in the flood-prone zones compared to the national baseline. The plot is on a log scale.

**Fig. 3:**
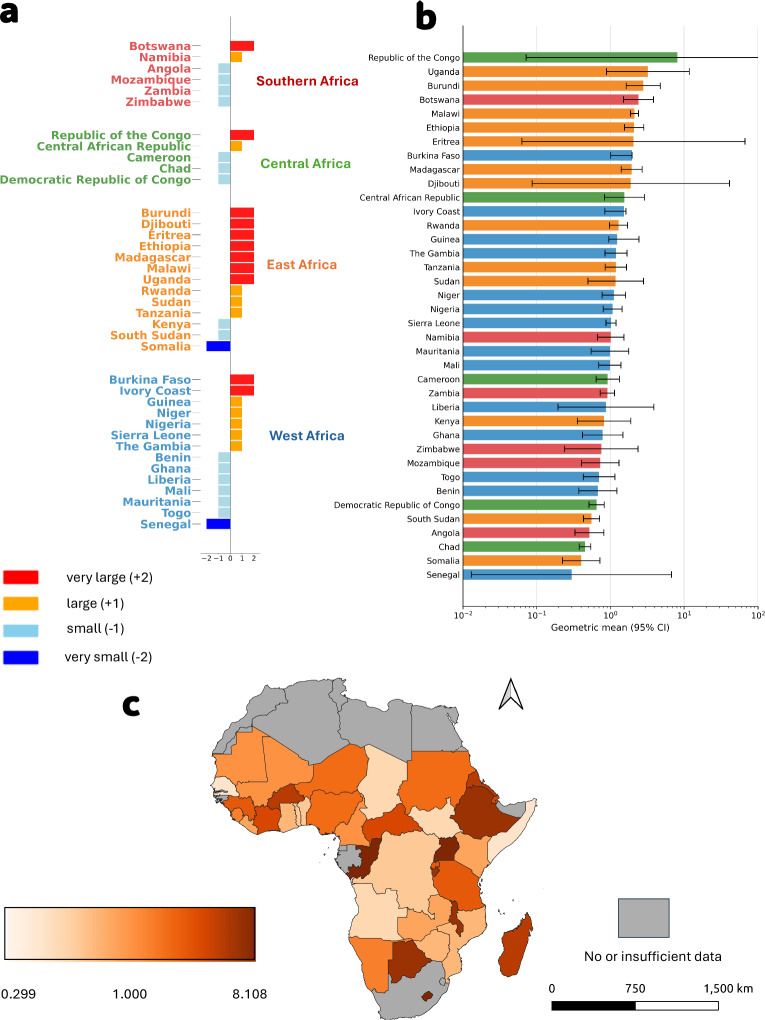
Malaria relative risk in the flood-prone zones. **a,** Quantitative analysis of the geometric mean RR associated with some classes. Based on the results across all countries, malaria risk shows a large increase in 8 countries and a large decrease in two countries. The CI can be for all the countries listed in Supplementary Table 1. **b,** Geometric mean of the RR by country, CI 95%. **c,** RR mean burden of 38 Sub-Saharan Africa countries.

**Malaria relative risk**. Using an empirical approach, we calculated the malaria relative risk (RR) to assess malaria burden in flood-affected zones relative to each country’s baseline (see Methodology). We conducted the analysis based on trends of RR for each event between 2000 and 2018 (Fig. 2). We computed the geometric mean number of flood events per country and per event between 2000 and 2018, based on RR. We then analysed the mean RR per country and region using the geometric mean of flood events (see Methodology). The country with the highest RR mean is Republic of the Congo, with a RR of 8.108 (95% confidence interval [CI]: 0.072, 909.050), followed by Uganda (RR = 3.225, 95% CI: 0.884, 11.769) and Burundi (RR = 2.797, 95% CI: 1.641, 4.767), (Fig. 3, Supplementary Table 1). The wide gap in the CI reflects the randomness of flood events temporal fluctuations. Flood occurrences and their impacts tend to vary significantly from year to year. This temporal randomness reduces the precision of the estimated mean values and leads to broader CIs, indicating greater uncertainty in some cases. In Central Africa, the Central African Republic is classified as large, and the Democratic Republic of Congo as very Large; all the other countries are classified as small. Countries with a very large RR mean are predominantly clustered in East Africa (Fig. 3), accounting for approximately 77% of all countries with high RR means. In West Africa, 43% of events fall within the small RR mean category. Meanwhile, in Southern Africa, 66% of countries are categorised within the small RR mean class.

**Fig. 4:**
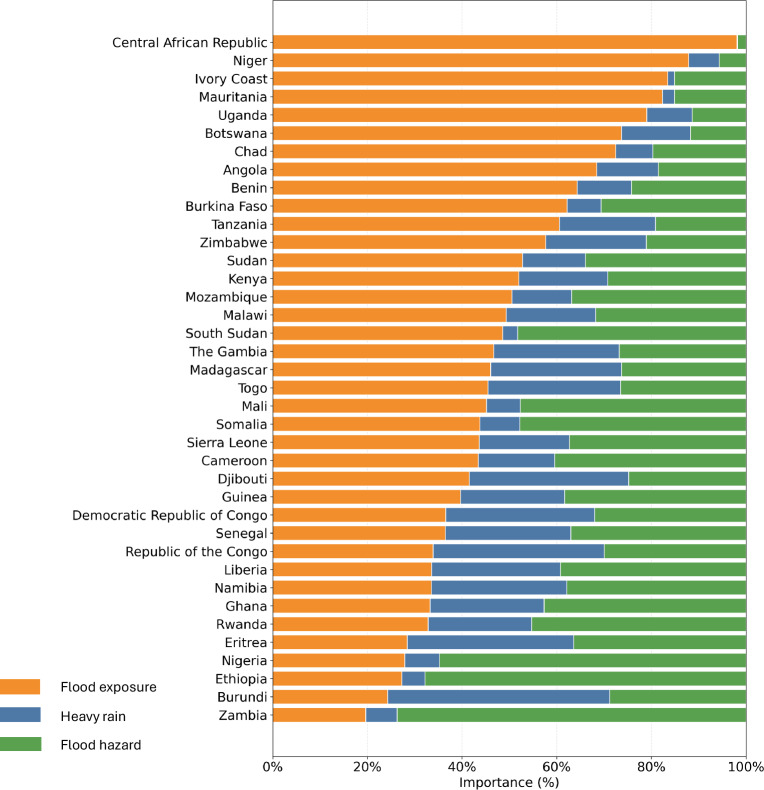
Drivers importance by country. The bar plot shows the hierarchical Bayesian model results and highlights the predominant covariate drivers for each country.

**Hierarchical Bayesian model**. Based on a hierarchical Bayesian model, we analysed the *Pf* impact on its covariates. We found that several dominant drivers contribute to *Pf* cases. These cases are primarily associated with flood exposure (65.7%), followed by flood size (29.0%), and, heavy rainfall (5.3%), (Fig. 4). The country with the highest association between the dependent flood exposure variable and *Pf* cases is the Central African Republic (Fig. 4).

## Discussion and conclusion

This study provides a continental-scale assessment across Africa, linking flood events to malaria burden in 38 sub-Saharan African countries over 18 years. By integrating satellite-based flood observations with *Pf* incidence data, we quantify the malaria risks associated with flood exposure in a region where both hydrological extremes and malaria burdens are intensifying. This work extends beyond prior country-level analyses by unveiling large-scale spatial and temporal heterogeneity in the flood–malaria relationship.

We estimate that approximately $\sim$12 million individuals exposed to flood events have been diagnosed with *Pf*. Our findings delineate geographic hotspots where flooding may play a pivotal role in shaping malaria risk. By integrating flood event information into malaria risk assessment, our analysis offers a new dimension to the GTS framework, enhancing its capacity to anticipate and mitigate malaria risk in flood-prone regions.

Our analysis uncovers the underlying mechanisms driving the coupled variability between flood occurrence and malaria burden across regional and national scales. Distinct regional signals emerge, with particularly elevated malaria risk and *Pf* cases observed in flood-prone zones of Eastern and Western Africa. Zooming in on individual countries, Uganda has the highest number of *Pf* cases. Uganda should get particular attention following flood events due to the elevated probability of a substantial malaria burden. The high RR and the second largest variability (with the wide CI) suggest that RR is not only greater than in other countries but also highly sensitive to flood magnitude and timing. This sensitivity likely reflects Uganda’s complex hydrotopography, including riverine and lacustrine systems that sustain mosquito breeding sites after inundation. The number of people exposed to flooding in Uganda will continue to increase in 2030^13,17^, which could increase the burden of malaria. We can observe the same pattern in the Republic of the Congo, which has the highest RR and the largest variability in the CI (Supplementary Tables 1 and 2).

East and West Africa are the regions with the highest malaria burden. The findings draw a parallel with WHO reports, highlighting these two regions as the primary malaria hotspots. The African countries, according to WHO data, continue to bear a disproportionately high share of the global malaria burden, with Nigeria, the Democratic Republic of the Congo, Niger, Ethiopia, Tanzania, and Mozambique accounting for the majority of cases. When comparing these findings with our own analyses, we observed that in this cohort, three countries exhibited an increasing RR over time. The stochastic nature of flood events complicates the identification of continuous global trends, yet localised patterns reveal essential insights. Our geospatial statistical model identifies flood exposure as the primary predictor of Pf incidence. This finding highlights the potential importance of populations exposed to hydrological events in malaria eradication strategies.

To conclude, we identified several hotspots and drivers of malaria risk in flood-prone zones, which may support malaria eradication efforts. This study, combined with analyses of past events, can inform future adaptation frameworks under climate change. Nevertheless, this study does not establish a direct causal relationship between flooding and malaria incidence. Our findings highlight key regions with elevated RR, and the resulting map can serve as a useful tool to identify where flooding coincides with a higher malaria burden. Our study supports malaria eradication strategies by informing policymakers about the geographic distribution of past vulnerable zones. This geospatial assessment can also be used to calibrate future projections. For future research, additional covariates could be included in the analysis. Vegetation and temperature data could be added, along with new socioeconomic indicators such as GDP per capita, poverty levels, and the Gini coefficient.

Several limitations should be acknowledged. The GFD dataset identifies 913 flood events using MODIS optical imagery, which cannot detect nocturnal or cloud-obscured floods, potentially omitting critical exposure events. The Malaria Atlas Project data carry uncertainties, especially in rural or conflict-affected regions where healthcare access and reporting coverage are inconsistent. The alignment of flood and population data introduces additional uncertainty due to spatial mismatches. Furthermore, the annual resolution of the malaria dataset prevents short-term (monthly) analysis, such as that demonstrated by He et al.^19^, which could better capture post-flood transmission dynamics. Finally, the absence of age-disaggregated data limits the ability to assess impacts on the most vulnerable group–children under five.

## Methods

Using geospatial data derived from flood maps provided by the GFD and annual malaria incidence maps from the Malaria Atlas Project *Pf*, we conducted a spatial overlay analysis to quantify the intersection between flood exposure and malaria risk. First, flood extent rasters were overlaid with the WorldPop^20^ dataset to estimate the population exposed to flooding. Population exposure is calculated by overlaying the flood inundation map with gridded population data. Second, we applied the same overlay method using the malaria incidence *Pf* maps for each flood event, allowing us to estimate the number of individuals diagnosed with *Pf* malaria residing within flood-affected areas.

**Flood hazard**. To get the flood maps flood hazard, we collected flood maps from the GFD for Sub-Saharan Africa between 2000 and 2018. This database is derived from satellite imagery (MODIS) with a resolution of 250 meters to estimate flood extent for 913 flood events between 2000 and 2018. In Sub-Saharan Africa, many flood events occur in border countries, and the dataset provides the flood events in this region, not by country. Thus, the flood pixel map was clipped to each country’s administrative boundaries, derived from OpenStreetMap, to delineate flood zones within each country at the time of the event. For each Sub-Saharan African country, we additionally collected data from the GFD on heavy rainfall (in millimetres) recorded during the flood period and zone.

**Flood exposure.** Flood exposure was analysed using WorldPop data version 1 (Global1). This dataset offers comprehensive and publicly accessible spatial demographic information, detailing the population count per cell with a resolution of 3 arcseconds. It provides global coverage from 2000 to 2020. Population estimates are available at resolutions of approximately 100 meters and 1 kilometre, along with data on individuals. We used data for the years 2000-05-10-15 to estimate the total number of people per grid square at five timepoints. The grid has a spatial resolution of 30 arc seconds (1 km at the equator). We aggregate the population grid to a 5x5 km resolution to match the malaria data. Using a zonal statistical^21^ approach, we overlay the flooded pixel map and population data to get the flood exposure.

**Malaria burden in flood zones.** We obtained data from the Malaria Atlas Project on the incidence of diagnosed Plasmodium falciparum. The dataset combines prevalence survey data and routine surveillance reports, along with a comprehensive set of geospatial covariates that characterise habitats suitable for *Anopheles mosquitoes*, the primary vectors of human malaria. The resulting maps cover all malaria-endemic countries and are produced annually at a 5 $\times$ 5 km resolution. During the study, we identified 20 anomalies (out of n = 492 flood events) in which the number of reported *Pf* cases within the flood zone exceeded the estimated population at risk. These inconsistencies may arise from the limited spatial granularity of the maps and the underestimation of population counts, as well as mismatches between the malaria case dataset and the population dataset or due to repeated infections. Our analyses take in count those anomalies with a sensitivity test (see Sensitivity section).

### Statistical analyses

**Relative risk ** For each year and for each country, we performed a relative risk ($RR_{t}$) to assess the incidence of malaria in the flood zone ($\textit{Pf}R_{\text {fe}}$) compare to the country baseline ($\textit{Pf}R_{\text {c}}$). This ratio reflects the malaria burden, adjusted by the proportion of the population exposed to floods (i.e, those living in flood zones) relative to the country’s total population. Using this method, we can observe a potential deviation from the baseline in the flood zone. This method is used in epidemiology and risk analysis to compare the probability of an event happening in one group versus another^22^. In this case, we compare the malaria burden in the flood zone to the country’s malaria burden (annually for each event). We use the method at the time of the event (*t*). The incidence in the country is divided by the total population of the country ($\textit{P}_{\text {tot}}$) and in the flood zone by the number of people flooded $\textit{E}_\textit{x}(\textit{F}_{\textit{x}})$, Eq. (1). A ${RR}_{it}>1$ indicates a positive percentage in the flood zone compared to the country, and ${RR}_{it}<1$ indicates a negative percentage.

1 $$\begin{aligned} RR_{t} = \frac{\textit{Pf}_{\text {fe}, t} / \textit{E}_\textit{x}(\textit{F}_\textit{x})_t}{\textit{Pf}_{\text {c}, t} / P_{\text {tot}, t}} \end{aligned}$$

We used the geometric mean to calculate the average of ($RR_{t}$) to provide a clear view of trends. The geometric mean averages the role of extreme floods and small floods. This statistical method is often used for ratios and percentages derived from values Eq. (2). Secondly, we calculated the CI 95% Eqs. (3) and (4):

2 $$\begin{aligned} \overline{RR}_t = \exp \left( \frac{1}{n} \sum _{i=1}^{n} \ln (RR_{t,i}) \right) \end{aligned}$$

3 $$\begin{aligned} \text {Lower 95\% CI} = \overline{RR}_t \cdot \exp \left( -z \cdot s_{\ln } \right) \end{aligned}$$

4 $$\begin{aligned} \text {Upper 95\% CI} = \overline{RR}_t \cdot \exp \left( z \cdot s_{\ln } \right) \end{aligned}$$

Where $s_{\ln }$ is the standard deviation of the log-transformed data, and $z \approx 1.96$ for large $n$. The final percentage change of RR for one country over the study period can be calculated as:

5 $$\begin{aligned} \Delta _c = (\overline{RR}_t - 1) \times 100 \end{aligned}$$

The $RR_{t}$ is divided into four categories: very large, large, small, and very small. Those four categories allow for the capture of the global tendency of the geometric mean for each country. Following the methodology of Kreibich et al.^23^, values of $RR_{t}$ above 50% are classified as very large; otherwise, they are classified as large. Similarly, values of ($RR_{t}$) below -50% are classified as very small; otherwise, they are classified as small.

**Sensitivity.** The elevated ratios observed in certain cases may partly reflect differences in the spatial scale of flooding. Smaller flood extents can produce higher relative risks even when fewer individuals are affected, as population exposure is confined to a more localised hazard footprint. To assess the sensitivity and robustness of the estimated ratios and to reduce potential bias, we performed a Monte Carlo simulation for each country and all $RR_{t}$ values, using 1,000 iterations of the variables in Eq. (1) (Supplementary Table 2).

**Bayesian hierarchical.** We analysed the relationship between environmental (flood size, rainfall intensity) and socioeconomic (number of people exposed to flooding) drivers and parasite burden using a Bayesian hierarchical framework based on a Gaussian process. This approach is widely used as an advanced spatial statistical method in epidemiology, as it accounts for the spatial and temporal structure of the data^24–26^. The response variable is *Pf* cases within the flood-affected zone. Covariates included flood exposure, mean precipitation (measured within the flooded region over the flood event window), and total flooded area. Mean precipitation within the flood zone and event window is obtained from the Global Flood Database. This framework enables the incorporation of spatial autocorrelation and provides a more accurate representation of the delayed effects of flooding on malaria transmission dynamics^27^.

## Supplementary Information

Supplementary Information 1.

Supplementary Information 2.

## Data Availability

Malaria data were obtained from the Malaria Atlas Project and are available at https://data.malariaatlas.org/maps?layers=. Population count data for Africa were obtained from the WorldPop dataset: https://hub.worldpop.org/doi/10.5258/SOTON/WP00004. Flood maps and heavy rainfall data were obtained from the Global Flood Database: https://global-flood-database.cloudtostreet.ai/. The processed results and analyses derived from the data can be found at https://zenodo.org/records/17535397

## Code availability

Python code (version 3.9.16) for statistical analysis is available on GitHub at https://github.com/jeremyEudaric/Flood_Malaria_burden.
