# Supplementary material for: Geospatial mapping of malaria risk in flood-prone zones of Sub-Saharan Africa
Source: Sci Rep. 2026 Jun 10;16:18002. doi: 10.1038/s41598-026-54811-7 (PMC13254309; doi:10.1038/s41598-026-54811-7)
Supplement: Supplementary file 2 — Supplementary Information 2. [file 41598_2026_54811_MOESM2_ESM.pdf]

Supplementary Table 1
Supplementary Table 2

Supplementary Table 1: Malaria relative risk. The table represents the geometric mean for each country based on the ratio for each flood event CI 95%.

	Mean RR	95% CI	Mean RR%	Trend	Anomalies	P value:
Angola	0.519	[0.331 ,0.815]	-48.1%	Small	0	0.0072
Benin	0.676	[0.373 , 1.225]	-32.4%	Small	0	0.1704
Botswana	2.412	[1.509 , 3.854]	141.2%	Very large	0	0.0021
Burkina Faso	1.974	[1.011, 1.486]	97.4%	Very Large	5	0.0274
Burundi	2.797	[1.641, 4.767]	179.7%	Very large	0	0.0011
Cameroon	0.922	[0.641, 1.325]	-7.8%	Small	3	0.6442
Central African Republic	1.551	[0.831, 2.896]	55.1%	Large	1	0.1225
Chad	0.453	[0.378, 0.543]	-54.7%	Small	0	0.0000
Democratic Republic of Congo	0.651	[0.513, 0.825]	-34.9%	Small	0	0.0010
Djibouti	1.890	[0.087, 40.986]	89.0%	Very large	0	0.4671
Eritrea	2.056	[0.063, 66.739]	105.6%	Very large	0	0.2312
Ethiopia	2.099	[1.553, 2.839]	109.9%	Very large	1	0.0000
Ghana	0.789	[0.420, 1.483]	-21.1%	Small	1	0.4180

Guinea	1.239	[0.953, 2.450]	23.9%	Large	1	0.2384
Ivory Coast	1.528	[0.842, 1.626]	52.8%	Very Large	2	0.0722
Kenya	0.821	[0.356, 1.896]	-17.9%	Small	0	0.6176
Liberia	0.870	[0.195, 3.890]	-13.0%	Small	0	0.7279
Madagascar	1.951	[1.408, 2.702]	95.1%	Very large	0	0.0019
Mali	0.986	[0.696, 1.395]	-13.4%	Small	1	0.9281
Malawi	2.124	[1.870, 2.412]	112.4%	Very large	0	0.0000
Mauritania	0.988	[0.549, 1.776]	-1.2%	Small	0	0.9626
Mozambique	0.730	[0.406, 1.311]	-27.0%	Small	0	0.2728
Namibia	1.014	[0.671, 1.531]	1.4%	Large	0	0.9450
Niger	1.118	[0.774, 1.613]	11.8%	Large	0	0.5211
Nigeria	1.077	[0.801, 1.448]	7.7%	Large	1	0.6090
Republic of the Congo	8.108	[0.072, 909.050]	710.8%	Very Large	2	0.1966
Rwanda	1.295	[0.977, 1.716]	29.5%	Large	0	0.0689

Senegal	0.299	[0.013, 6.772]	-70.1%	Very small	0	0.3802
Sierra Leone	1.021	[0.873, 1.195]	2.1%	Large	0	0.6989
Somalia	0.403	[0.225, 0.723]	-59.7%	Very small	0	0.0045
South Sudan	0.556	[0.433, 0.715]	-44.4%	Small	0	0.0001
Sudan	1.182	[0.496, 2.815]	18.2%	Large	0	0.6857
Tanzania	1.189	[0.852, 1.660]	18.9%	Large	0	0.2937
The Gambia	1.195	[0.847, 1.686]	19.5%	Large	1	0.2238
Togo	0.704	[0.430, 1.154]	-29.6%	Small	2	0.1275
Uganda	3.225	[0.884, 11.769]	222.5%	Very large	0	0.0730
Zambia	0.912	[0.729, 1.141]	-8.8%	Small	0	0.3981
Zimbabwe	0.755	[0.240, 2.377]	-24.5%	Small		0.6138

Supplementary Table 3: Monte Carlo simulation of the malaria relative risk with 1000 iterations. For the Monte Carlo simulation, we are performing 1000 iterations using the parameter values from the RR equations.

	Mean RR	95% CI	Standard deviation
Angola	0.751	[0.444, 1.184]	0.190
Benin	0.879	[0.542, 1.213]	0.169
Botswana	2.922	[1.907, 4.308]	0.627
Burkina Faso	1.947	[1.697, 2.237]	0.137
Burundi	5.557	[2.142, 11.584]	2.876
Cameroon	0.969	[0.889, 1.054]	0.042
Central African Republic	1.583	[1.328, 1.884]	0.141
Chad	0.484	[0.411, 0.569]	0.040
Democratic Republic of Congo	0.774	[0.594, 0.975]	0.099
Eritrea	2.126	[1.563, 2.704]	0.399

Ethiopia	2.470	[1.868, 3.069]	0.307
Ghana	0.630	[0.527, 0.747]	0.056
Guinea	1.261	[1.097, 1.443]	0.090
Ivory Coast	1.596	[1.398, 1.814]	0.105
Kenya	1.399	[0.848, 2.048]	0.306
Liberia	0.964	[0.448, 1.456]	0.230
Madagascar	1.984	[1.728, 2.267]	0.140
Mali	1.116	[0.989, 1.250]	0.068
Malawi	2.137	[1.946, 2.359]	0.104
Mauritania	1.247	[0.726, 1.914]	0.311
Mozambique	1.051	[0.785, 1.302]	0.132
Namibia	1.241	[0.890, 1.654]	0.192

Niger	1.317	[0.929, 1.740]	0.206
Nigeria	1.128	[1.037, 1.221]	0.046
Republic of the Congo	3.771	[3.078, 4.741]	0.426
Rwanda	1.450	[1.142, 1.789]	0.171
Senegal	0.922	[0.589, 1.160]	0.153
Sierra Leone	1.024	[0.945, 1.105]	0.042
Somalia	0.656	[0.367, 0.995]	0.163
South Sudan	0.639	[0.478, 0.862]	0.101
Sudan	3.087	[1.468, 5.024]	0.924
Tanzania	1.455	[1.158, 1.771]	0.151
The Gambia	1.234	[1.036, 1.601]	0.160
Togo	0.775	[0.533, 1.069]	0.136
Uganda	26.10	[7.541, 50.610]	10.62
Zambia	1.005	[0.825, 1.207]	0.097

Zimbabwe	1.392	[1.030, 1.869]	0.217
